# A Feasibility Study on Proton Range Monitoring Using ^13^N Peak in Inhomogeneous Targets

**DOI:** 10.3390/tomography8050193

**Published:** 2022-09-15

**Authors:** Md. Rafiqul Islam, Mehrdad Shahmohammadi Beni, Akihito Inamura, Nursel Şafakattı, Masayasu Miyake, Mahabubur Rahman, Abul Kalam Fazlul Haque, Shigeki Ito, Shinichi Gotoh, Taiga Yamaya, Hiroshi Watabe

**Affiliations:** 1 Graduate School of Biomedical Engineering, Tohoku University, Sendai 980-8579, Japan; 2 Institute of Nuclear Medical Physics, AERE, Bangladesh Atomic Energy Commission, Dhaka 1349, Bangladesh; 3 Division of Radiation Protection and Safety Control, CYRIC, Tohoku University, Sendai 980-8578, Japan or; 4 Department of Physics, City University of Hong Kong, Tat Chee Avenue, Kowloon Tong, Hong Kong; 5 Nuclear Safety Security Safeguard Division, Bangladesh Atomic Energy Regularity Authority, Dhaka 1207, Bangladesh; 6 Department of Physics, University of Rajshahi, Rajshahi 6205, Bangladesh; 7 Mirai Imaging Inc., Fukushima 970-1153, Japan; 8 GO Proton Japan Inc., Tokyo 169-0074, Japan; 9 National Institutes for Quantum Science and Technology, Chiba 263-8555, Japan

**Keywords:** proton range monitoring, Monte Carlo method, inhomogeneous targets, proton therapy, positron emission tomography, PET

## Abstract

Proton irradiations are highly sensitive to spatial variations, mainly due to their high linear energy transfer (LET) and densely ionizing nature. In realistic clinical applications, the targets of ionizing radiation are inhomogeneous in terms of geometry and chemical composition (i.e., organs in the human body). One of the main methods for proton range monitoring is to utilize the production of proton induced positron emitting radionuclides; these could be measured precisely with positron emission tomography (PET) systems. One main positron emitting radionuclide that could be used for proton range monitoring and verification was found to be ^13^N that produces a peak close to the Bragg peak. In the present work, we have employed the Monte Carlo method and Spectral Analysis (SA) technique to investigate the feasibility of utilizing the ^13^N peak for proton range monitoring and verification in inhomogeneous targets. Two different phantom types, namely, (1) ordinary slab and (2) MIRD anthropomorphic phantoms, were used. We have found that the generated ^13^N peak in such highly inhomogeneous targets (ordinary slab and human phantom) is close to the actual Bragg peak, when irradiated by incident proton beam. The feasibility of using the SA technique to estimate the distribution of positron emitter was also investigated. The current findings and the developed tools in the present work would be helpful in proton range monitoring and verification in realistic clinical radiation therapy using proton beams.

## 1. Introduction

Radiation therapy using charged particles has gained lots of attraction in the battle against cancer. In recent comparisons between conventional and proton radiation therapy techniques, it was found that proton irradiations would lead to a lesser dispersed dose to non-targeted organs [1], given that the ultimate goal of radiation therapy would be to eradicate cancer cells while keeping healthy and normal cells intact. Using charged particles such as protons in radiation therapy, it would be possible to achieve a rather high dose conformity by exploiting the Bragg peak [2], which is the location that charged particles would deposit highest amount of dose in the target. The location of Bragg peak has a particular importance mainly due to the sharp increase of dose deposition in the target material. Therefore, it is pertinent to determine this location (i.e., Bragg peak) rather precisely, to enhance killing of cancer cells and limiting the dose to normal healthy cells.

Considering the spatial sensitivity of the Bragg peak location and in turn variations in the maximum dose deposition, geometrical and chemical inhomogeneities play an important role in these variations [3,4,5,6,7,8]. An example of such inhomogeneous targets is organs in the human body. In fact, the human body is composed of many organs, each having distinct geometrical features and chemical compositions. Therefore, such geometrical and chemical variations at the interface of tissues or organs would alter the dose distribution of protons; this would in turn lead to changes in the Bragg peak location and make the proton range monitoring and verification tedious. Several techniques for proton range monitoring were proposed, including: proton radiography and tomography [9]; ionoacoustics [10]; Secondary Electron Bremsstrahlung (SEB) [11]; and Prompt Gamma Imaging (PGI) [12]. In addition to these methods, auto-activation Positron Emission Tomography (PET) [13,14] technique is another notable technique, mainly explored after the imaging of proton induced positron emitting target fragments [15]. This method is non-invasive, which would be highly desirable for realistic clinical applications. The deposited dose in a target element (i.e., volume) could be measured by detecting annihilation radiation emitted as a result of generated positron emitting radionuclides (such as ^15^O, ^11^C and ^13^N) produced through nuclear reactions between protons and elements in the target [16].

One particularly important positron emitting radionuclide is ^13^N that is mainly produced through ^16^O(p,2p2n)^13^N nuclear reaction, with a low threshold energy of 5.660 MeV. Considering the low threshold and rather substantial yield of ^13^N producing nuclear reaction, it was found to be useful for proton range monitoring and verification [17]. Previously, we have investigated the use of this nuclear reaction and the generated ^13^N peak for proton range monitoring and verification in homogeneous targets both numerically [18] and experimentally [19]. In our previous works, we have also developed a spectral analysis (SA) approach to detect ^13^N production from dynamic PET data and succeeded in rendering ^13^N distribution proximal to the Bragg peak in homogenous phantom targets. Considering the effect of inhomogeneities on the proton range monitoring and verification, it would be pertinent to investigate the feasibility of using ^13^N for proton range monitoring and verification in inhomogeneous targets.

In the present work, we have extended our previously developed tools [18,19] for investigating the feasibility of using ^13^N for proton range monitoring and verification in inhomogeneous target phantoms. The Particle and Heavy Ion Transport code System (PHITS) (https://phits.jaea.go.jp/ (accessed on 14 September 2022)) Monte Carlo (MC) package was used. Two different phantom types, namely, (1) ordinary slab and (2) MIRD anthropomorphic human models, were used. The employment of such highly inhomogeneous targets would provide useful information on the actual effectiveness and feasibility of using ^13^N peak for proton range monitoring and verification. We strongly believe the developed tools, as well as the obtained results, would be useful for future developments in the field of proton range monitoring using PET-based systems.

## 2. Materials and Methods

### 2.1. The Monte Carlo Method

In the present work, the PHITS MC package was used to model the interaction and transport of proton in the modelled inhomogeneous phantoms. The JENDL-4.0/HE cross-section data were used to explain the interaction of protons with matter. The PHITS also used the INC-ELF (intra-nuclear cascade with emission of light fragment) model for nuclear reactions with energy more than 20 MeV. In addition, the production of proton induced positron emitting radionuclides was computed using the microscopic transport model (JAM) and the JAERI quantum molecular dynamics (JQMD) model. The reaction cross section for ^13^N production as a result of proton irradiation can be found from Ref. [16]. Namely, two different tallies, (1) energy deposition and (2) production, were used. The employed T-product tally was set to a 3D *xyz* mesh that covered the entire region of the phantom to count the generated positron emitting radionuclides. The T-product tally would score the number of generated radionuclides over the modelled target, both at their place of generation and places that they would travel. The generation and transport of positron emitting radionuclides were considered automatically using the employed PHITS MC package. The decay of these species was also considered according to the distribution that was estimated using the MC method. The energy deposition tally was used to determine the energy deposition of protons in the modelled inhomogeneous phantoms. The production tally was used to score the amount of generated proton induced positron emitting radionuclides in the modelled inhomogeneous phantoms, as incident protons propagate through matter.

### 2.2. The Ordinary Slab Phantom Model

**Figure 1:**
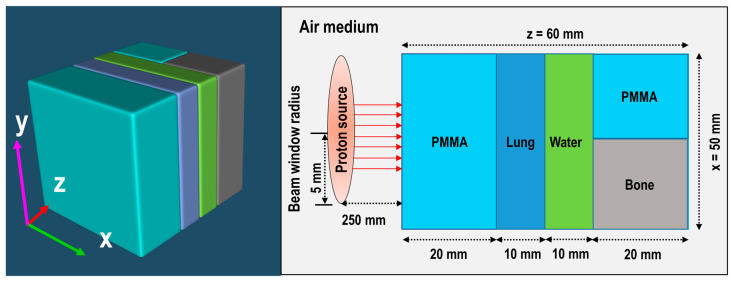
Schematic representation of modelled ordinary slab phantom with dimensions.

The first inhomogeneous model is the ordinary slab phantom, composed of five different domains. Each domain in the modelled ordinary slab phantom has different geometry and chemical compositions; this resembles an inhomogeneous target for the purpose of this work. The ordinary slab phantom consisted of 20 mm PMMA (polymethyl methacrylate), 10 mm lung equivalent (inflated), 10 mm water, and 20 mm bone equivalent (cortical bone slab) and another 20 mm of PMMA for the last domain. The densities and the material compositions used in the ordinary slab phantom are shown in Table 1. The densities and material composition were taken from Refs. [3,4,5]. The modelled ordinary slab phantom is shown schematically in Figure 1. The height of the modelled ordinary slab phantom was set to be 50 mm for all five domains. The modelled ordinary slab phantom was inspired from the previously reported structure in Ref. [8].

The arrangement of the five domains shown in Figure 1 were used to ensure geometrical and chemical composition variations along the incident proton beam. The proton beam was modelled as a disk source, emitting radiation in a pencil-like manner, having radius of 5 mm. The energy of incident protons was set to be at 80 MeV and 70–80 MeV with 1 MeV interval energy modulation, for obtaining pristine and spread-out Bragg peak (SOBP), respectively. The source of protons to target distance was set to be 250 mm along the incident proton beam. A total of 10^9^ incident protons were launched from the source, to minimize the statistical uncertainties associated with MC computations.

### 2.3. The MIRD Anthropomorphic Phantom

**Figure 2:**
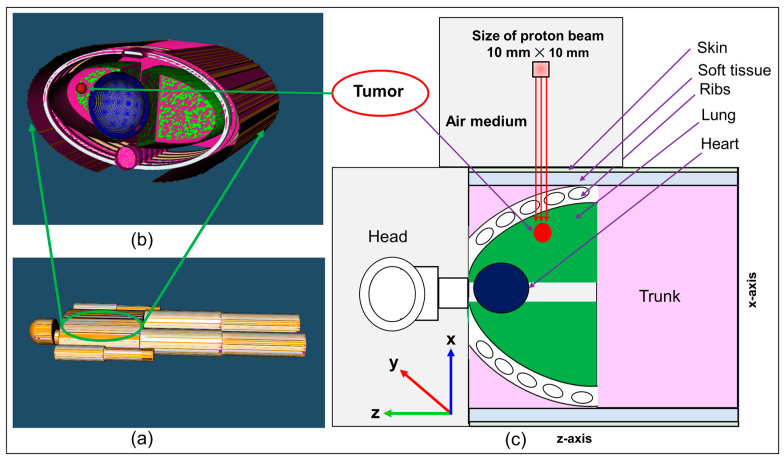
The modelled MRID anthropomorphic phantom with tumor location marked. The irradiation of the spherical tumor placed at left lung is also shown with a 10 × 10 mm^2^ field size.

The Medical Internal Radiation Dose Committee (MIRD) anthropomorphic phantom was used to investigate the effect of inhomogeneities of human tissues on the obtained ^13^N peak location and its offset with the Bragg peak. The deposited proton dose and distribution of proton induced positron emitting radionuclides were determined in a spherical tumor placed in the left lung of the MIRD anthropomorphic phantom. The diameter of the spherical tumor was set to be 10 mm. The composition of the tumor was set to be same as soft tissue. The modelled MIRD anthropomorphic phantom, tumor location, and irradiation zone, are shown schematically in Figure 2. The densities and the material composition for the modelled MIRD anthropomorphic phantom were taken from Ref. [20] and summarized in Table 2. Regarding the other elements (shown in the last column of Table 2), the stable isotopes (i.e., mass number shown in the periodic table) of these elements were considered.

The tumor located in the left lung of the modelled MIRD anthropomorphic phantom was irradiated with 80 MeV monoenergetic incident proton beam (see Figure 2). The irradiation field size set to be a 10 × 10 mm^2^ squared shaped source. The beam was perpendicular to the tumor volume along the negative *y*-axis. A total of 10^9^ incident protons were launched from the source, to minimize the statistical uncertainties associated with MC computations.

### 2.4. Tallies and Post-Processing of Results

For both ordinary slab (see Figure 1) and MIRD anthropomorphic phantom (see Figure 2), the dose deposition versus depth and production of positron emitting radionuclides (i.e., ^15^O, ^11^C and ^13^N) were tallied along the incident proton beam. The T-deposit and T-product tally option provided by PHITS MC package were used for both pristine and SOBP beam types. The tallied results were in fact normalized per primary source particles, which is the default of every MC package. Considering the half-life for the positron emitting radionuclides (i.e., ^15^O, ^11^C and ^13^N), the obtained results were converted into activity. These were then computed over a time grid from 1 to 55 min. The obtained output data were then analyzed using our previously developed PyBLD software developed using Python programming language (http://www.rim.cyric.tohoku.ac.jp/software/pybld/pybld.html (accessed on 14 September 2022)). The generated images were visualized using the AMIDE (A Medical Image Data Examiner) version 1.0.4 software (link: http://amide.sourceforge.net (accessed on 14 September 2022)) [21,22]. The PyBLD and the AMIDE software were used for image construction from the generated PHITS output data and image visualization, respectively. In addition, the one-dimensional (1D) dose versus depth and activity profiles were obtained; this provided information regarding the location of the activity and its distribution. Time dependent activities were obtained for 0, 15, 20, 30, and 55 min for ^15^O, ^11^C, and ^13^N positron emitting radionuclides. The obtained 1D, two-dimensional (2D) plots provided information about the location of the Bragg peak and those from ^15^O, ^11^C, and ^13^N positron emitting radionuclides. The generated images that provide indication of possible practical implementation in real PET systems, were constructed using the data obtained by MC method by employing mesh based tallies available by default in the PHITS MC package.

### 2.5. The Spectral Analysis (SA) Method

Similar to our previous works on homogeneous targets [18,19], we have employed our previously developed SA technique to quantify the amount of produced ^15^O, ^11^C, and ^13^N positron emitting radionuclides in the modelled target phantoms. The SA was initially developed for kinetic analysis of PET tracer [23]. The term “spectral analysis” indicates a single input/output model used for the data quantification in dynamic PET studies. The impulse response function (IRF) of the compartmental models used in PET can be resolved as the analytical sum of exponentials. This approach allows a tracer’s tissue time-activity curves (TACs) to be described in terms of an ideal subset of kinetic components chosen from a much larger set. It usually consists of convolution integrals of the input function with decaying exponentials, allowing the selected components to be connected to a suitable compartmental model for the system under investigation. Considering each radioisotope to be one component at time *t*, and its input function *A(t)*, then the time activity curve of each voxel *C_v_(t)* which is expressed by the following equation:

(1) $$C_{v}\left(t\right)=\overset{M}{\underset{j=1}{\sum }}A\left(t\right)⊗\alpha_{j}e^{-\beta_{j}t}\;\;\;\;\;$$

where *α_j_* and *β_j_* are assumed to be real-valued and nonnegative. The ⊗ is convolution operation. *M* represents the maximum number of terms to be included in the model that was set to 1000. The original SA approach for PET kinetic analysis utilizes “concentration” as the main parameter, however, in our approach, relative changes are the main focus and therefore the unit would be arbitrary. The values of *β_j_* are predetermined and fixed in order to cover an adequate range of spectral values. For each *β_j_*, *A(t)* ⊗*e^−βjt^* (impulse response function (IRF)) is precalculated while we assumed *A(t)* is impulse input function (1 at time 0 and 0 at time >0). The values of αj are estimated from the time-activity curves by a nonnegative least squares (NNLS) procedure. When the IRFs are precalculated, then the estimation sets of *α_j_* are entirely linear, and SA can instantly find groups of *α_j_* without repeating the computation. For each voxel *v*, the numerical *S_v_* values were then computed using the following equation with a threshold of *β* > 1.5 to eliminate the background region:

(2) $$S_{v}\left(t\right)=\underset{j=1}{\sum }\alpha_{j}\beta_{j}$$

Considering Equation (2), it needs to be noted that the upper limit of summation will be varied for each voxel. The ranges of the *β* were different for different positron emitting radionuclides. In order to extract the shorter half-life or in other words larger *β* (0.693/T_1/2_) radionuclides such as; ^15^O and ^13^N, a range of 0.005 (threshold) to 0.006 (betacut) s^−1^, and 0.001 to 0.002 s^−1^ were used, respectively. Whereas, for longer half-life or smaller *β* (0.693/T_1/2_) radionuclide such as; ^11^C, the range of *β* 0.0005 to 0.0006 s^−1^ was used.

### 2.6. Region of Interest (ROI) Used by SA Technique

**Figure 3:**
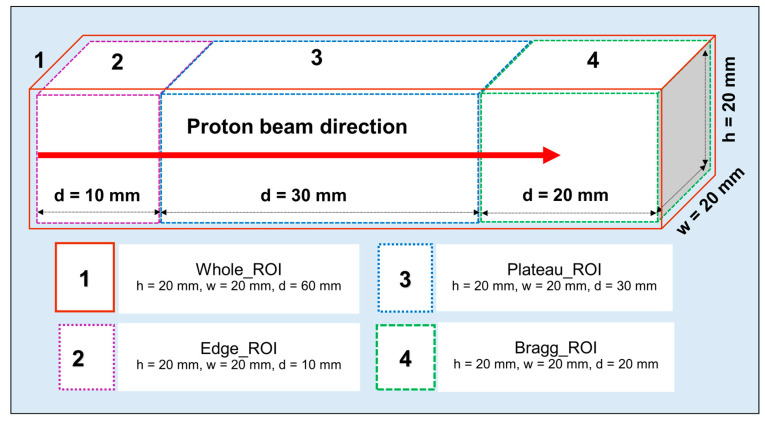
An illustration of the four regions of interest (ROIs) and their height (*h*), width (*w*) and depth (*d*) values used in SA technique.

The SA method was used to extract the positron emitting radionuclides (^15^O, ^11^C, and ^13^N) from the obtained MC results. We refer interested readers to our previous work for a step-by-step scheme [19]. For the ordinary slab phantom, the SA technique was applied to the 40 frames (from 15 to 55 min; considering 15 min after proton irradiation). Then, the time activity curve was calculated from the 3D data set of 40 frames, each having 1 min interval over 40 min. These were scored using four regions of interest (ROIs): (1) whole (d = 60 mm); (2) edge (d = 10 mm); (3) plateau (d = 30 mm); and (4) Bragg region (d = 20 mm), as shown in Figure 3. Using these data, a single voxel image for each ROI was generated. The obtained results could be used to confirm the presence of proton induced positron emitting radionuclides in every region shown in Figure 3. In the reported results, the obtained ^13^N positron emitting radionuclide was displayed as “SA_^13^N”. The SA approach was applied to the MIRD anthropomorphic phantom in the same way as slab inhomogeneous phantom study for four regions of interest (ROIs): (1) whole (d = 80 mm); (2) edge (d = 10 mm); (3) plateau (d = 40 mm); and (4) Bragg region (d = 30 mm), but with a different depth since the Bragg peak would be produced at a deeper location. Using these data, we generated time course of averaged value within the defined ROI region.

## 3. Results and Discussion

**Figure 4:**
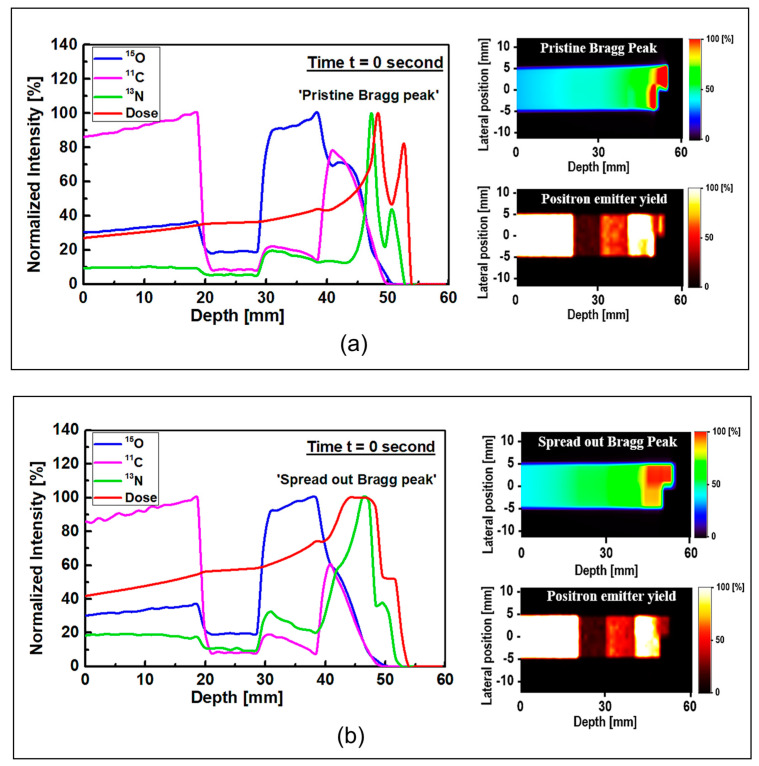
The 1D and 2D profiles of the simulated depth-dose of protons and production of positron emitting radionuclides (^15^O, ^11^C and ^13^N) in ordinary slab phantom for (**a**) pristine and (**b**) SOBP incident proton beams.

The results of 1D and 2D dose versus depth and production of positron emitting radionuclides in ordinary slab phantom are shown in Figure 4. The results from pristine and SOBP proton beams were shown. The results were normalized so the comparison between the dose-depth profile and profile from positron emitting radionuclides can be seen clearly. From the results in Figure 4, it can be seen that the Bragg peak sharply splits into two peaks; this is due to the interface between the PMMA and bone equivalent materials placed at the end of ordinary slab phantom (see Figure 1). The difference between the stopping power of protons in PMMA and bone equivalent material is the main reason for observing this phenomenon. The projected range of protons at the material interface, which has two different chemical compositions, will change based on the changes in the stopping power of protons. A denser medium, such as bone material that has higher stopping power, would lead to lower proton range and for the case of PMMA, the proton range would be higher than that of bone material. On the contrary, for the SOBP proton beam, the splitting of the Bragg peak (i.e., the Bragg peak formation at the interface between PMMA and bone material) is not notably sharp mainly due to the extended Bragg peak region as the result of spreading, which essentially resembles multiple Bragg peaks. Interestingly, for both pristine and SOBP incident proton beams, the effect of material interface and changes in its chemical composition on the projected range of protons can be clearly seen, as shown in 2D contour plots in Figure 4. From the dose shown in the results in Figure 4, these values are normalized according to the maximum value. In addition, the SOBP was modelled using energy modulation at which energy bin having similar energy as of pristine beam would have lower number of protons (i.e., lower fluence) as that of pristine beam; this mixture of incident proton energies, the energy deposition and fluence of protons in entire PMMA and bone domain will not be the same, given that proton would stop at a lower depth in bone, mainly due to its composition and density which leads to a higher stopping power of protons when compared to PMMA. Considering these, the relative dose in bone would be lower than that of PMMA. The 1D profiles were obtained along the modelled inhomogeneous target. In realistic scenarios, the measurements would be performed over all regions and cannot be only limited to only one single region with specific material.

Considering the generated positron emitting radionuclides, the generated ^15^O positron emitting radionuclides was found to be higher in the water filled region of the modelled ordinary slab phantom, which is located at ~30–40 mm depth (see Figure 4). The production of ^15^O radionuclides is through the ^16^O(p,pn)^15^O nuclear reaction channel, which has a threshold of 16.79 MeV. It needs to be noted that the interaction of protons with ^16^O also has the ability to undergo ^16^O(p,3p3n)^11^C nuclear reaction, which has the threshold of 27.50 MeV. Considering the location of the water filled region in the ordinary slab phantom and energy loss of protons after passing through multiple different regions before reaching water filled region, the production of ^11^C positron emitting radionuclides in the water filled region is not a dominant nuclear reaction channel. Therefore, the amount of ^15^O radionuclides would be higher compared to ^11^C radionuclides in the water filled region; this can be seen from the unnormalized results shown in Appendix A.

The production of ^11^C radionuclide in the PMMA filled region was found to be higher than other regions in the modelled ordinary slab phantom for both pristine and SOBP proton beams. The generation of ^11^C radionuclide is mainly through the ^12^C(p,pn)^11^C nuclear reaction channel which has a relatively higher threshold energy of 20.61 MeV. The PMMA filled region has relatively higher ^12^C content and is located in a location closest to the incident proton beam. Therefore, the chemical composition of PMMA and higher energy of protons in the PMMA filled region would enhance the production of ^11^C positron emitting radionuclides, hence a dominant peak can be observed in the obtained results shown in Figure 4.

The most important positron emitting radionuclide that is found to be generated in the modelled phantoms is ^13^N. The ^13^N positron emitting radionuclides would be generated as a result of ^14^N(p,pn)^13^N and ^16^O(p,2p2n)^13^N nuclear reactions having threshold energy of 11.44 and 5.660 MeV, respectively. Furthermore, considering the energy loss of protons as the results of their propagation through target material, the energy of protons decreases with depth. Considering the presence of lower energy protons at higher depths and the presence of ^14^N and ^16^O elements in the modelled ordinary slab phantom, more ^13^N radionuclides would be generated at higher depths along the path of incident protons; this can be seen in the results shown in Figure 4. The ^16^O(p,2p2n)^13^N nuclear reaction is inclusive of ^16^O(p,α)^13^N [16].

The ^13^N peak would be located behind the Bragg peak; this is due to the energy threshold of ^16^O(p,2p2n)^13^N nuclear reaction that prevent the generation of ^13^N radionuclides when proton energy decreases significantly at locations adjacent (i.e., very close) to the Bragg peak. Comparing the offset distance between the Bragg peak and the ^13^N peak, an offset distance of ~1 mm was obtained for both peaks shown in Figure 4a. The obtained offset value is in a good agreement with our previous work on irradiation of homogeneous phantoms [18]. For the SOBP proton beam results shown in Figure 4b, the 13N peak is within the same ~1 to ~2 mm offset distance that we have previously reported [18]. However, since the SOBP is broad, the ^13^N peak would be inside the SOBP region, see Figure 4b. Interestingly, the generated ^13^N peak in the SOBP region (see Figure 4b) is broader than that of pristine Bragg peak (see Figure 4a), mainly due to the proton beam energy modulation that was used to achieve a spread-out Bragg peak. Since the production positron emitting radionuclides are highly dependent on the incident energy of primary radiation (i.e., protons), it would be safe to conclude that changes in the beam shape through energy modulation is strongly correlated to the production and distribution of positron emitting radionuclides. The stopping power of protons in bone is higher than that of PMMA and, therefore, protons would have a longer travel path in PMMA when compared to bone material; this leads to a broader distribution of energy in PMMA material and in turn the distribution of ^13^N would get broader. Considering these, a higher difference between the Bragg peak and the generated ^13^N peak is observed in PMMA. This phenomenon is in fact important when dealing with inhomogeneous targets.

**Figure 5:**
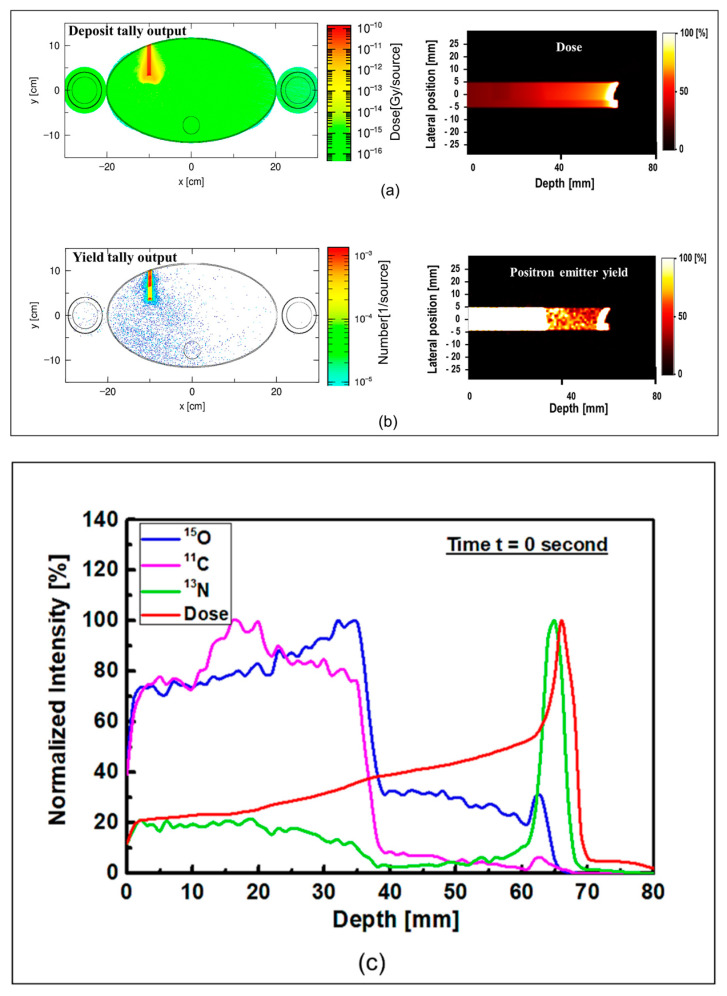
The energy deposition (top-left) and production of positron emitting radionuclides (top-right) obtained in the modelled MIRD anthropomorphic phantom. The 2D distribution of: (**a**) dose deposition of protons; (**b**) distributions of positron emitting radionuclides determined along the incident proton beam track; and (**c**) the distribution of deposited dose and positron emitting radionuclides versus depth in the body of MIRD anthropomorphic phantom.

The results of the proton dose deposition and production of positron emitting radionuclides (^15^O, ^11^C, and ^13^N) in the modelled MIRD anthropomorphic phantom are shown in Figure 5. The distribution of deposited dose and positron emitting radionuclides along the incident proton beam within the body of anthropomorphic phantom were visualized (see Figure 5). The 2D contour plot of dose and positron emitting radionuclides were obtained and shown in Figure 5a,b. The 1D distribution of deposited dose and positron emitting radionuclides versus depth in the body of MIRD anthropomorphic phantom is shown in Figure 5c.

The dose deposition and production of positron emitting radionuclides in the body of MIRD anthropomorphic phantom (see Figure 5) shows the fact protons interact along the beamline toward the tumor region. As a result of proton interaction and subsequent production of positron emitting radionuclides through nuclear interaction, positron emitting radionuclides would be generated. The number of these generated radionuclides found to be highest along the proton beam, mainly due to higher number of nuclear interactions induced by protons. Interestingly, some of these generated radionuclides would be dispersed in other regions of modelled MIRD anthropomorphic phantom.

The 2D contour plot of deposited proton dose and positron emitting radionuclides shown in Figure 5a,b, respectively, proves the fact that protons would produce positron emitting radionuclides along their path in the modelled MIRD anthropomorphic phantom. The offset in the final part of the 2D distribution shows the effect of beam interaction with spherical tumor, as can be seen from the curved end of simulated dose (see Figure 5a) and positron emitter yield (see Figure 5b). The results of the positron emitting radionuclides obtained in the body of modelled MIRD anthropomorphic phantom and 2D contour plots (i.e., Figure 5b) represent the total production of all positron emitting radionuclides (^15^O, ^11^C, and ^13^N).

In order to distinguish the contribution of each positron emitting radionuclide, the distribution of deposited dose by incident protons and individual tallied positron emitting radionuclide versus depth were obtained and shown in Figure 5c. The distribution of ^15^O and ^11^C positron emitting radionuclides was found to be highest at lower depths, mainly due to higher ^16^O and ^12^C contents in the soft tissue and lungs which enhances the ^16^O(p,pn)^15^O, ^16^O(p,3p3n)^11^C, and ^12^C(p,pn)^11^C nuclear reactions. Another important reason would be proton energy, which would be higher at lower depth and in turn satisfy the energy threshold for ^15^O and ^11^C producing nuclear reactions. The produced ^13^N peak is close to the Bragg peak (~1 mm) mainly due to its low energy threshold. The offset between the Bragg peak and the generated ^13^N peak was found to be about ~1 mm, where the Bragg peak and the generated ^13^N peak are located at 66 and 65 mm, respectively. As discussed previously, the obtained offset value is in good agreement with our previously reported data [18].

The ultimate goal of the present feasibility study is to apply the developed methods for realistic PET data obtained after proton irradiations. In realistic PET measurements of the signal produced by the positron emitting radionuclides (^15^O, ^11^C, and ^13^N), the detected signal will originate from different radionuclides. In addition, the inhomogeneities of the target could in fact complicate the task of separating the signals from different positron emitting radionuclides. Therefore, it would be pertinent to deconvolute these to obtain the ^13^N peak for proton range monitoring and verification. To achieve this, the SA technique was applied to the simulated data for ordinary slab and MIRD anthropomorphic phantoms.

**Figure 6:**
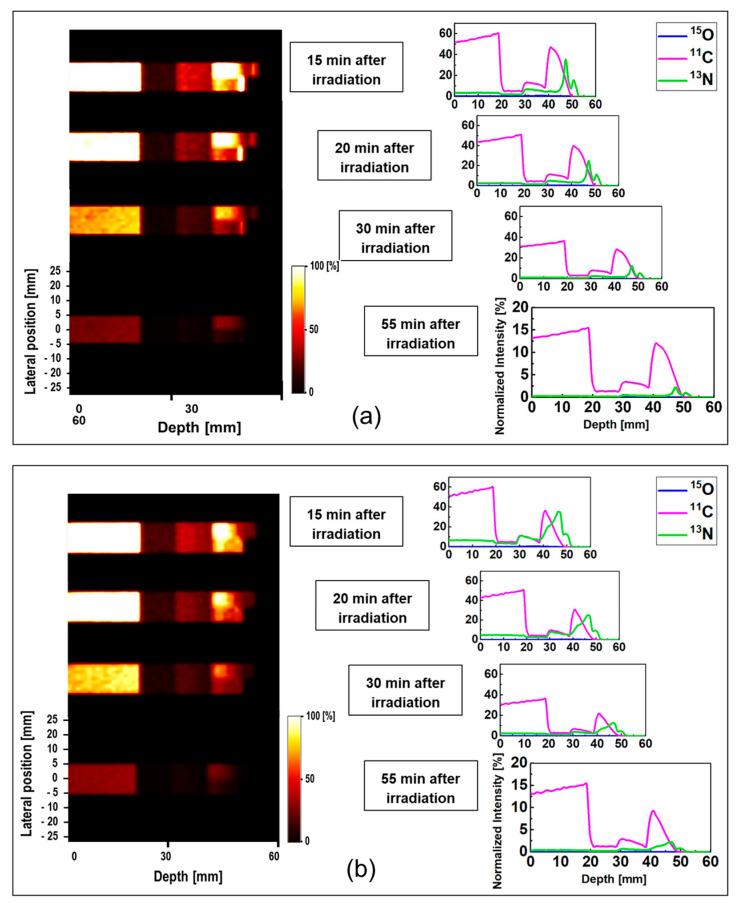
The 2D and 1D time-course activity analysis in the time range of 15 to 55 min for ^15^O, ^11^C, and ^13^N positron emitting radionuclides in the ordinary slab phantom irradiated with (**a**) pristine and (**b**) SOBP beams.

The obtained data for ordinary slab and MIRD anthropomorphic phantoms was converted into time-course images; this can be helpful for lengthy data acquisition process in realistic PET experiments. The 1D and 2D time-course activity in the time range of 15 to 55 min for ^15^O, ^11^C, and ^13^N positron emitting radionuclides in the ordinary slab phantom is shown in Figure 6. The results for the pristine and SOBP beams are shown in Figure 6a,b. The *y*-axis of the 1D profiles was labeled as the relative intensity. Similar scaling factors as shown in Figure 4 and Figure 5 were used. In the time-course profile data of both pristine and SOBP beams, the relatively short-lived ^15^O spectrum with half-life of 2.03 min almost disappeared. The spectrum of ^11^C radionuclide was found to be dominant in both pristine and SOBP beams in the considered time frame of 15 to 55 min; this is mainly due to its long half-life of 20.33 min which would in fact remain detectable for rather a long time. The spectrum of ^13^N was found to be visible at higher depths and stayed detectable even up to 55 min after the proton irradiation. The ^13^N radionuclide has a half-life of 9.93 min which makes it detectable for quite some time after it fully decays.

**Figure 7:**
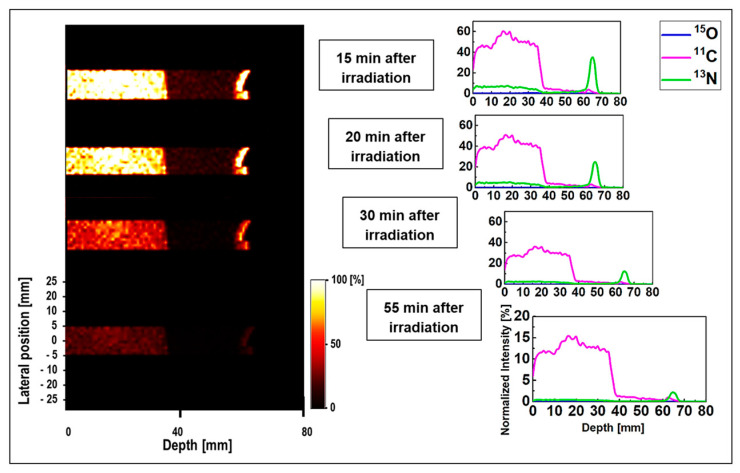
The 2D and 1D time-course activity analysis in the time range of 15 to 55 min for ^15^O, ^11^C, and ^13^N positron emitting radionuclides in the MIRD anthropomorphic phantom irradiated 80 MeV monoenergetic proton beam.

The 1D and 2D time-course activity in the time range of 15 to 55 min for ^15^O, ^11^C, and ^13^N positron emitting radionuclides in the MIRD anthropomorphic phantom is shown in Figure 7. The *y*-axis of the 1D profiles was labeled as the relative intensity. Similar scaling factors as shown in Figure 4 and Figure 5 were used. Similar to the time-course activity of ordinary slab phantom shown in Figure 6, the relatively short-lived ^15^O positron emitting radionuclides were vanished 15 min after the irradiation and was not visible for longer time frames (i.e., *t* > 15 min). The spectrum of ^11^C radionuclide was also found to be dominant in MIRD anthropomorphic phantom, as discussed this is mainly due to its long half-life of 20.33 min. However, the interesting difference in the spectrum of ^11^C in ordinary slab and MIRD anthropomorphic phantoms is the shape of this spectrum. The shape of ^11^C spectrum is rather more complex in the ordinary slab phantom mainly due to presence of multiple domains with different sizes and material compositions. However, for the MIRD anthropomorphic phantom, the geometrical changes along the incident proton beam in more subtle given that protons experience lesser geometrical and chemical composition changes along their track when passing through thin layer of skin, soft tissue, and ribs, to reach the tumor region. Similarly, the spectrum of ^13^N was found to be visible at higher depths and stayed detectable up to 55 min after the proton irradiation (see Figure 7). These results show that it would be possible to detect ^13^N peak for proton range verification in realistic clinical applications by deconvoluting signals from different positron emitting radionuclides using our proposed SA technique.

**Figure 8:**
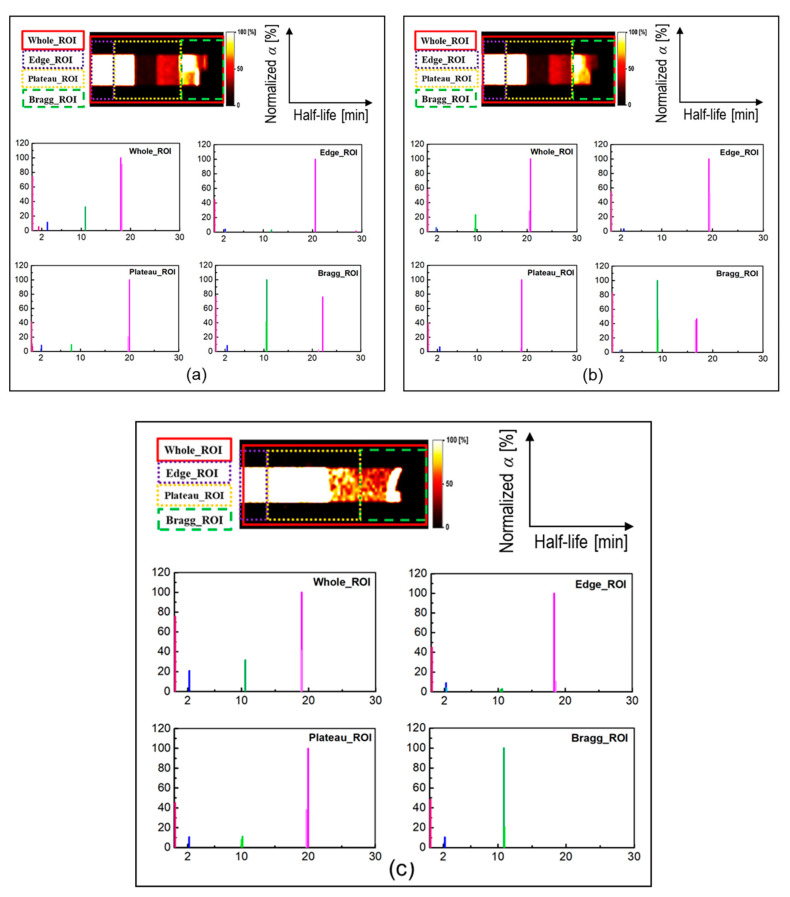
The results of spectral analysis (SA) for different ROIs. Pink shows no radioisotope component, blue shows ^15^O, magenta shows ^11^C and green shows ^13^N positron emitting radionuclides components for: (**a**) ordinary slab phantom irradiated with pristine beam; (**b**) ordinary slab phantom irradiated with SOBP beam; and (**c**) for MIRD anthropomorphic phantom irradiated with 80 MeV monoenergetic beam.

The SA technique was applied to the time-course dataset (from 15 to 55 min after irradiation) to predict the half-life of each positron emitting radionuclides present in the realistic PET measurements; this would be particularly useful for experimental investigations to identify and distinguish different radionuclides generated as a result of proton irradiation. The obtained results from the SA approach at different ROIs introduced in Figure 3 are shown in Figure 8a–c. The *x*- and *y*-axis, shown in Figure 8, represent the extracted half-life and normalized counts, respectively. The contribution from ^11^C positron emitting radionuclides that have relatively longer half-life compared to other generated radionuclides, found to be dominant in whole, edge, and plateau regions. The contribution from ^15^O and ^13^N radionuclides in whole, edge, and plateau regions, were found to be smaller than that of ^11^C. However, the contribution from ^13^N positron emitting radionuclides in the Bragg peak ROI was found to be dominant. This trend was observed for both ordinary slab (for both pristine and SOBP beams) and MIRD anthropomorphic phantoms. The present method is not only limited to the ROIs shown in Figure 3; in fact, users can apply this technique to any desired region in their offline PET experiments. The distribution was calculated using value of each voxel present in the considered region. The component of zero half-life implies background count or offset and does not represent any realistic radionuclides.

**Figure 9:**
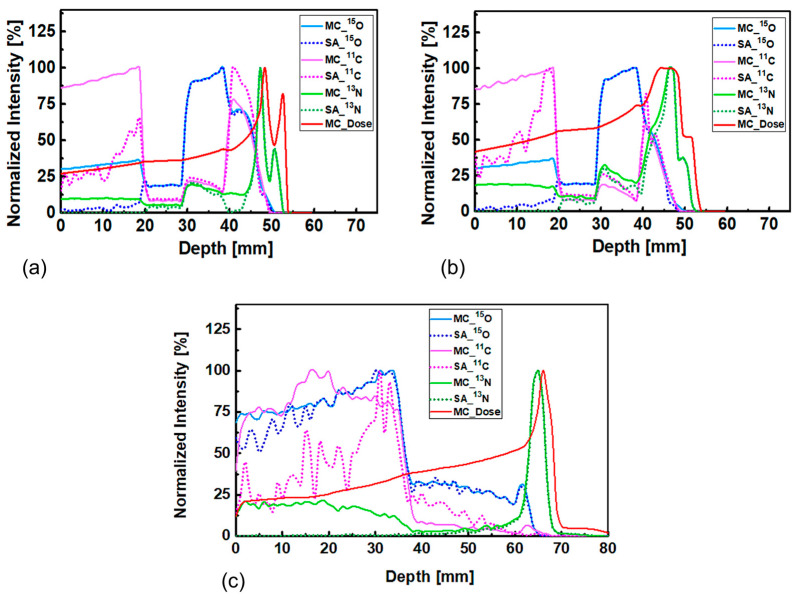
The activity of positron emitting radionuclides obtained from MC and SA methods for: (**a**) ordinary slab phantom irradiated with pristine beam; (**b**) ordinary slab phantom irradiated with SOBP beam; and (**c**) for MIRD anthropomorphic phantom irradiated with 80 MeV monoenergetic beam.

The results shown in Figure 9a,c show the comparison between the activity of positron emitting radionuclides obtained from MC and SA methods. The comparison between the pristine and SOBP beams for ordinary slab phantom is shown in Figure 9a,b, respectively. This comparison between the activity of positron emitting radionuclides obtained from the MC and SA methods for MIRD anthropomorphic phantom is shown in Figure 9c. The estimated results from the MC and SA methods are in good agreement, particularly for the ^13^N positron emitting radionuclide that would be used for proton range monitoring and verification. Some degree of discrepancies between the activity of positron emitting radionuclides obtained from the MC and SA methods can be observed at lower depths; this is mainly due to very close contribution from positron emitting radionuclides (^15^O, ^11^C, and ^13^N) at the entrance region where incident proton beam initially interacts. The SA method experiences difficulties in accurately separating these at the entrance region, however the estimations from the SA approach improve at higher depths. From the results shown in Figure 9, it can be concluded that the predicted ^13^N radionuclide peak using the SA method is close to the peak determined by the MC method, which in turn would be close to the actual Bragg peak (~1 mm). Therefore, the SA method found to be feasible to predict activity distribution of positron emitting radionuclides when applied to the ordinary slab and MIRD anthropomorphic phantoms.

The average relative error obtained from our MC computations for deposited proton dose in ordinary slab phantom and MIRD anthropomorphic phantom found to be less than 0.002 and 0.006, respectively. The average relative error for obtained ^13^N production found to be less than 0.0835 and 0.17 in ordinary slab phantom and MIRD anthropomorphic, respectively.

It is important to note that different radionuclides would have a different range in tissues, and this could in fact influence real PET measurements. In addition, at the interface zone between two different materials, the PET system with higher resolutions would be useful when trying to determine the emission from different positron emitting radionuclides. The optimal PET system for the range monitoring of proton therapy must have: (1) high sensitivity to detect activated positron emitters; (2) handling dynamic data to process using the SA; (3) flexible geometry to position PET detectors near to any irradiated areas; and (4) resistance against high radiation background. The dual-head positron emission mammography system is one candidate to fulfill these conditions.

## 4. Conclusions

The present study showed the feasibility of utilizing the ^13^N peak for proton range monitoring and verification in inhomogeneous targets. Two different phantom types, namely, (1) ordinary slab and (2) MIRD anthropomorphic phantom models, were used. Two different incident proton beams, namely, (1) pristine and (2) SOBP, were used in the irradiation of ordinary slab phantom. A monoenergetic 80 MeV proton beam was used to irradiate the modelled MIRD anthropomorphic phantom. We have found that the generated ^13^N peak in such highly inhomogeneous targets (ordinary slab and MIRD anthropomorphic phantoms) is close to the actual Bragg peak (~1 mm), when irradiated by incident proton beam. The feasibility of employing our previously developed SA technique was also investigated. The activity of positron emitting radionuclides obtained from the MC and SA methods for ordinary slab phantom irradiated with pristine and SOBP beams and MIRD anthropomorphic phantom, were found to be in good agreement. The developed tools and reported results would be useful for future developments in the field of proton therapy. In future works, we aim to perform cyclotron irradiation experiments with inhomogeneous slab phantoms and compare the results with the present theoretical investigations.

## Figures and Tables

**Table 1:** Densities and materials composition (shown in weight percent) of the ordinary slab phantom.

Material	Density (g·cm^−3^)	^1^H	^12^C	^14^N	^16^O	^23^Na	^24^Mg	^31^P	^32^S	^40^Ca
Water	1.000	11.10	-	-	88.90	-	-	-	-	-
PMMA	1.180	8.050	59.99	-	31.96	-	-	-	-	-
Lung equivalent	0.2600	10.30	10.50	3.100	74.90	0.2000	-	0.2000	0.3000	-
Bone equivalent	1.8500	3.400	15.50	4.200	43.50	0.1000	0.1000	10.30	0.3000	22.50

**Table 2:** Densities and materials composition (shown in weight percent) of the MIRD anthropomorphic phantom.

Material	Density (g·cm^−3^)	^1^H	^12^C	^14^N	^16^O	Other Elements
Skin	1.090	10.00	20.40	4.200	64.50	0.2Na, 0.1P, 0.2S, 0.3Cl, 0.1K
Soft tissue	1.030	10.50	25.60	2.700	60.20	0.1Na, 0.2P, 0.3S, 0.2Cl, 0.2K
Heart	1.050	10.40	13.90	2.900	71.80	0.1Na, 0.2P, 0.2S, 0.2Cl, 0.3K
Blood	1.060	10.20	11.00	3.300	74.50	0.1Na, 0.1P, 0.2S, 0.3Cl, 0.3K, 0.1Fe
Lung	0.2600	10.30	10.50	3.100	74.90	0.2Na, 0.2P, 0.3S, 0.3Cl, 0.2K
Ribs	1.410	6.400	26.30	3.900	43.60	0.1Na, 0.1Mg, 6.0P, 0.3S, 0.1Cl, 0.1K, 13.1Ca

**Figure A1:**
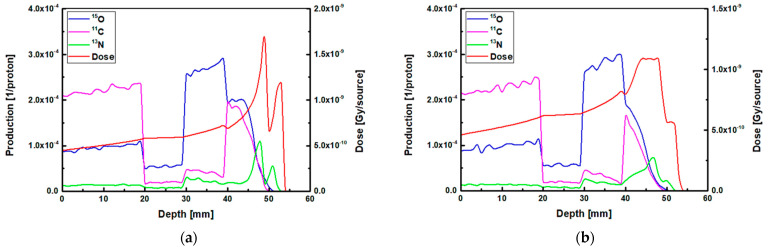
The unnormalized 1D profiles of the simulated depth-dose of protons and production of positron emitting radionuclides (^15^O, ^11^C, and ^13^N) in ordinary slab phantom for (**a**) pristine and (**b**) SOBP incident proton beams.

## Data Availability

The data can be downloaded from: https://figshare.com/articles/figure/PET-based_proton_range_monitoring_using_a_SA_approach_in_an_inhomogeneous_target/16577471 (accessed on: 14 September 2022).

## Appendix A

The unnormalized 1D profiles of the simulated depth-dose of protons and production of positron emitting radionuclides (^15^O, ^11^C, and ^13^N) in ordinary slab phantom for pristine and SOBP incident proton beams are in shown in Figure A1a,b, respectively. These are unnormalized results that were shown in Figure 4 of the present paper.
